# Identification of bioactive compounds from Fraxinus angustifolia extracts with anti- NADH oxidase activity of bovine milk xanthine oxidoreductase

**DOI:** 10.3906/biy-1810-26

**Published:** 2019-04-05

**Authors:** Nadjia AHMANE, Dina ATMANI-KILANI, Nassima CHAHER, Karima AYOUNI, Meriem RAHMANI-BERBOUCHA, Grégory DA COSTA, Nadjet DEBBACHE-BENAIDA, Tristan RICHARD, Djebbar ATMANI

**Affiliations:** 1 Laboratory of Applied Biochemistry, Faculty of Nature and Life Sciences, University of Béjaïa , 06000 , Algeria; 2 University of Bordeaux, Research Unit of Oenology , Villenave d'Ornon , France

**Keywords:** Fraxinus angustifolia, NADH oxidase, xanthine oxidoreductase, phenolics, hyperuricemia

## Abstract

Fraxinus angustifolia leaves and bark are used in traditional medicine against various inflammatory-related pathologies incumbent to reactive oxygen species (ROS) generation by the NADH oxidase activity of enzymes such as xanthine oxidoreductase (XOR). This study was designed to investigate the in vitro and in vivo inhibitory activities of this enzyme by Fraxinus angustifolia extracts. The leaf organic phase of ethyl acetate (LFA) and its bark aqueous counterpart (BFA) showed the strongest anti-NADH oxidase activity in vitro (IC50 = 38.51 and 42.04 µg mL-1, respectively). They consequently suppressed superoxide generation both enzymatically (53% and 19%, respectively) and nonenzymatically (34% and 19%, respectively). These results were corroborated in vivo, with high antiNADH oxidase potential of the leaves and bark extracts (75.32% and 51.32%, respectively) concomitant with moderate hypouricemic activities (36.84% and 38.59%, respectively). Bio-guided fractionation led to the identification, by LC-DAD-MS/MS, of esculin and calcelarioside in bark and kaempferol glucoside in leaves as the main compounds responsible for the anti-NADH oxidase activity of XOR. These results plead in favor of the use of F. angustifolia as a source of potentially interesting therapeutic substances.

## 1. Introduction

Xanthine oxidase (XO) and xanthine dehydrogenase (XDH) are interconvertible forms of the same enzyme, known as xanthine oxidoreductase (XOR). In a mammalian fresh tissue, XOR exists under the XDH form, which is NAD-dependent and produces primarily NADH at the FAD site (Waud and Rajagopalan, 1976; Hattori, 1989). However, this form is easily converted to an O2- dependent type (XO) during the procedures of extraction and purification. Both forms of the enzyme show NADH oxidase activity, with generation of ROS, but XDH is somewhat more effective in this respect (Atmani et al., 2005). In milk, the physiological function of xanthine oxidase has long been a puzzle, but it turned out to play an antimicrobial defensive role in the neonatal gut because of ROS generation (Harrison, 2005) . The secretion of milk fat globules is another useful task of the enzyme in a process dependent on the enzyme protein rather than on its enzymic activity (Harrison, 2006). In liver and intestine, ROS species are directly produced by XOR, with uric acid as its end product, whereas they are generated secondarily by XDH, as the enzyme produces primarily the reduced β-nicotinamide adenine dinucleotide (NADH) (Sanders et al., 1997; Vorbach et al., 2002). In this case, an increase is observed in NADH concentration and the generation of O2- and H2O2 is greatly amplified (Maia et al., 2007). It has been demonstrated that these ROS species are involved in the genesis of pathologies such as alcoholic hepatotoxicity (Teplova et al., 2017) and ischemia-reperfusion injury (Granger et al., 2001) . Additionally, simple upregulation of XOR activity, irrespective of XDH/XO ratios, could well be triggered either by hypoxia or by pro-inflammatory agents, implying a strong link of the enzyme with inflammation (McCord and Roy, 1982; Cantu-Medel lin and Kelley, 2013) . Hyperuricemia could also be generated by a high XO/XDH activity, causing an accumulation of uric acid crystals in joints with the ultimate development of gouty arthritis characterized by an inflammatory leukocyte response (Haskard and Landis, 2002) . Hence, the inhibition of XO/XDH will contribute to the healing of this disease.

Numerous studies were directed towards seeking powerful natural inhibitors of xanthine oxidase (Berboucha et al., 2009) , but much less attention was given to the attenuation of its NADH oxidase activity. In this context, we adopted a novel approach by aiming to study the inhibitory effect of Fraxinus angustifolia (Oleaceae) extracts on the NADH oxidase activity of XOR. This plant was selected mainly because of its antiinflammatory usage in traditional medicine in Algeria (Beloued, 1998) ; in particular, its leaves have antirheumatismal properties while the bark is indicated against passive hemorrhages, gout, cholelithiasis and especially against intermittent fevers (Djerroumi and Nacef, 2004; Kostova and Iossifova, 2007). These medicinal virtues are greatly valorized by rural populations and may be attributed to the wealth of the plant in polyphenols such as secoiridoids, phenylpropanoids, and lignin glucosides (Kong et al., 2002).

Previously, we reported the strong antioxidant potential of F. angustifolia extracts (Atmani et al., 2009; Ayouni et al., 2016) , including the inhibition of the xanthine oxidase activity of XOR (Berboucha et al., 2010) , as well as their antiinflammatory (Medjahed et al., 2016) and antidiabetic (Lowman et al., 1983) activities. To the best of our knowledge, the ability of F. angustifolia extracts to inhibit the NADH oxidase activity of XOR has not been studied thus far. The present study also aimed to identify the specific molecules responsible for its anti-NADH oxidase activity in order to validate the traditional use of this plant in Algeria against inflammation. For that purpose, we conducted a bio-guided fractionation against the NADH oxidase activity of XOR.

## 2. Materials and methods

### 2.1. Chemicals

The buffer used, ethanol, ethyl acetate, chloroform, and sodium chloride (NACL) were purchased from Biochem, Chemopharma (USA). Xanthine oxidase (XO), xanthine, potassium oxonate, B-Nicotinamide adenine dinucleotide reduced disodium salt hydrate (NADH), DL-dithiothreitol (DTT), luteolin, sephadex G-25 gel, and dimethyl sulfoxide (DMSO) were purchased from Sigma-Aldrich (France). B-Nicotinamide adenine dinucleotide (NAD+) and diphenyleneiodonium (DPI) were obtained from Alfa Aesar (Germany) and acetic acid from Sigma-Aldrich (Germany). Ferrous sulfate and sodium carboxymethyl cellulose (CMC), rutin, tannic acid, and vanillic acid were purchased from Sigma-Aldrich (USA). Uric acid was obtained from Spinreact (Spain), and cytochrom C and phanazine methosulfate (PMS) were obtained from Fluka Biochemica (USA). Nitroblue tetrazolium (NBT), tris (hydroxymethyl) aminomethane hydrochloride (TrisHCl), and albumin bovine serum (BSA) were obtained from Biochem Chemopharma (France). Aluminum chloride anhydrous (AlCl3) was obtained from Biochem Chemopharma (Canada). EDTA disodium salt was purchased from Prolabo (EU) and sodium dodecyl sulphate (SDS) from Panreact (Spain). Folin-Ciocalteu’s reagent was obtained from Chim-Oza (France), hydrochloric acid (HCL) from Organics (Germany), and potassium chloride (KCl) from Prolabo (France). Sodium carbonate (Na2Co3) and ferric chloride (FeCl3) were obtained from Biochem (Montreal, Quebec).

### 2.2. Plant material

Leaves and barks of F. angustifolia were harvested in summer from the forest of Azru N’Bechar located in the Province of Amizour, Department of Bejaia (Northeastern Algeria), then dried at room temperature and ground to ifne powder (diameter < 63 µm) using an electric mill (Ika laboratechnik, Staufen, Germany). Plant material was identified by Professor Hacène Abdelkrim according to a listed voucher specimen (O/n◦59) in the herbarium of ENSA (Ecole Nationale Supérieure Agronomique), ElHarrach (Algiers, Algeria).

### 2.3. Animals

Albino male mice (18–25 g) were obtained from the Center of Research and Development (CRD SAIDAL, Algiers, Algeria). They were housed in cages and maintained on a 12-h light/dark cycle, at 25 °C, with constant humidity. Animals were handled according to the recommendations of the International Ethics Committee (Directive of the European Council 86/609/EC).

### 2.4. Extraction and fractionation

Extraction of polyphenols was performed using a previously described procedure (Atmani et al., 2009) .Briefly, the ground powders of leaves and barks were separately macerated in ethanol (95%) (1/4; w/v) for 24 h to obtain two crude extracts which were then dried. The leaf and bark ethanolic extracts were further subjected to fractionation in ethyl acetate and water (1/3/1; w/v/v), thereby yielding two separated fractions (ethyl acetate and aqueous). Two equal amounts of the ethyl acetate fraction were further fractionated using chloroform and water (1/3/1; w/v/v). The obtained subfractions were tested for their potential inhibition of the NADH oxidase activity of XOR.

### 2.5. Determination of total phenols, tannins, and flavonoids

The determination of total phenols in the extracts of the leaves and barks of F. angustifolia was carried out using standard procedure (Lowman and Box, 1983) . The reaction mixture was obtained with 2.5 mL of a solution of plant extract (0.1  mg mL−1) boiled in methanol, 25  mL of distilled water, 1.5  mL of sodium carbonate (Na2CO ) 3 (200 g L−1), and 0.5 mL (1 N) of Folin–Ciocalteu’s reagent. The mixture was allowed to stand for 60 min at room temperature, after which the absorbance was recorded at 750  nm. Total phenols were deduced from a standard curve and expressed in mg vanillic acid equivalents per gram of dry plant powder (mg VAE g−1).

The content of tannins in plant extracts was determined spectrophotometrically using the bovine serum albumin (BSA) precipitation method (Hagerman and Butler, 1978) .Solutions of BSA (2 mL) and tannic acid (1 mL) or extract dissolved in ethanol were mixed. After incubation for 15 min for tannic acid and 24 h for the extract at 4 °C, the mixtures were centrifuged at 3000 × g for 15  min. The resulting pellet was dissolved in 4 mL of SDS solution/ TEA (5% triethanolamine added to 1% SDS), followed by the addition of 1 mL of FeCl3. After 15 min of incubation, the absorbance was recorded at 510 nm against a blank. The content in tannins was calculated from the calibration curve using tannic acid as standard and expressed in mg tannic acid equivalent/g of extract (mg TAE g−1).

The flavonoid content in plant extracts was determined by a colorimetric method based on the formation of ammonium chloride (AlCl3) complex (Maksimović et al., 2005). The reaction mixture containing 10 mL (1 mg mL−1) of extract, 2 mL of distilled water, and 5 mL of AlCl3 was incubated for 10 min, after which the absorbance was measured at 430 nm. The content in flavonoids was computed from a standard curve using rutin and expressed in mg rutin equivalent/g of extract (mg RE g−1).

### 2.6. Bio-guided assays

### 2.6.1. Preparation of the dehydrogenase form of XOR

Xanthine oxidase was purified from bovine milk according to a previously described method (Sanders et al., 1997; Atmani et al., 2005) using centrifugation and column chromatography. The XDH form was obtained through reversible reduction of oxidized XO sulfhydryl groups by incubation with 10 mM dithiothreitol (DTT) for 2 h at 37 °C and then filtered through a small G-25 column.

### 2.6.2. XDH inhibitory activity

Xanthine dehydrogenase inhibitory activity, using NADH as substrate, was determined spectrophotometrically at 340 nm by following NADH disappearance under aerobic conditions every second for 3 min (Atmani et al., 2005) .The reaction mixture contained 1760 µL of phosphate buffer (20 mM, pH 7.0) and EDTA (0.1 mM), 200 µL of NADH solution (1 mM) and 20 µL of extract (100 µg mL−1) dissolved in DMSO or reference compounds (rutin and esculin). The reaction was initiated by the addition of 20 µL of XDH (1 U) solution. Diphenyleneiodonium (DPI) was also tested in the same conditions. The inhibitory activity of XDH was calculated as follows: % inhibition = [1− [Δ at / Δ Ac]] × 100, where ΔAt and ΔAc are the variations in absorbance of the test solution with and without the extract or reference drug, respectively.

### 2.6.3. Lineweaver–Burk plots

Extracts with high XDH-inhibitory activity were selected for performing the Lineweaver–Burk plot analysis in order to determine their mode of inhibition. This kinetic study was carried out in the absence or presence of either extracts or rutin with various concentrations of NADH (25, 50, 75, 100, and 125 µM). Kinetic parameters (Vmax and Km) were also determined.

### 2.6.4. Superoxide anion scavenging activity

The scavenging activity towards superoxide anion radical was determined according to the method of Liu et al. (1997). Superoxide anion was generated nonenzymatically by the phenazinemethosulfate-nicotinamide adenine dinucleotide (PMS-NADH) system based on the reduction of nitroblue tetrazolium (NBT). Superoxide anion was generated in 3 mL of Tris-HCl buffer (100 mM, pH 7.4) containing 0.75 mL of NBT (300 μM), 0.75 mL of NADH (936 μM), and 0.3 mL of extract (100 μg mL−1). The reaction was initiated by the addition of 0.75 mL of PMS (120 μM) to the mixture. After 5 min of incubation at room temperature, the absorbance was measured at 560 nm. The superoxide anion scavenging activity was calculated according to the following equation: % scavenging = [(A₀ − A₁) / A₀ × 100], where A0 and A1 are the absorbencies of the solution in the absence and presence of the extract, respectively.

### 2.6.5. Superoxide anion generation by NADH-xanthine dehydrogenase activity

Superoxide production was determined in terms of the reduction of cytochrome C (25 µM) and calculated by using an absorption coefficient of 21 mM −1 Cm−1, with NADH as the reducing substrate. The reaction mixture in a total volume of 1 mL contained 50 mM potassium phosphate buffer (pH 7.4), NADH (100 µM), and cytochrome C (25 µM). A concentration of 100 µg mL−1 of plant extract was added into the mixture. The reaction was initiated by the addition of XDH (25 mU mL−1) and monitored at 550 nm for 10 min.

### 2.7. UHPLC-MS analysis

LC–MS analyses were performed on an UHPLC 1290 series apparatus from Agilent Technologies (Santa Clara, CA, USA) connected to an Esquire LC-ESI-MS/MS from Bruker Daltonics (Bremen, Germany). The column used was a C18 reversed-phase ZORBAX Eclipse Plus column from Agilent Technologies (2.1 × 100 mm, 1.8 µm, 10 cm). A solution of 2 mg mL−1 of F. angustifolia extract was prepared in methanol/water (50/50), shaken vigorously, filtered, and injected directly into the HPLC system. The flow of solvent was 0.4 mL min −1 with an injection volume of 1 µL (solvent system of 0.1% (v/v) formic acid-water (A), 0.1% (v/v) formic acid-acetonitrile (ACN) (B)). The separation was conducted using the following gradient: solvent B 0 min at 10%, 1.7 min at 10%, 3.4 min at 20%, 5.1 min at 30%, 6.8 min at 30%, 8.5 min at 35%, 11.9 min at 60%, 15.3 min at 100%, 17.0 min at 100%, and 17.3 min at 10%. The absorbance was measured at two wavelengths as 280 and 360 nm.

### 2.7.1. Preparative HPLC chromatography

Pure compounds were obtained by preparative HPLC on a Smartline system from Varian TM (Smartline, Pump 1000, Manager 5000, Detector UV K-2600, Berlin, Germany). The chromatographic parameters were optimized for the best resolution and sensitivity. The extracts were prepared at a concentration of 200 mg mL−1 in methanol/ water (50/50). Peak shape analyses were performed using a column of Kinetex 5VXB-C18 (150 × 21.2 mm) with a gradient of acidified water (0.1% TFA; solvent A)/ acidified ACN (0.1% TFA; solvent B); the solvent flow rate was 20 mL min−1 for an injection volume of 250 µL. The separation was conducted using the following gradient: solvent B 0 min at 20%, 5 min at 20%, 30 min at 40%, 31 min at 100%, 37 min at 100%, and 45 min at 20%.

### 2.8. Hyperuricemia model in mice

The experimental animal model of hyperuricemia was established using a uricase inhibitor (potassium oxonate, 250 mg kg−1b.w), as previously described (Wang et al., 2010) .Oxonate, the extracts of plant, and diphenyleneiodonium (DPI) as positive reference were dissolved or dispersed in CMC (0.8%). Food, but not water, was withdrawn from the animals 1 h prior to drug or extract administration.

### 2.8.1. Drug administration

Mice were divided into categories I and II composed respectively of normal and hyperuricemic mice. Each category was divided into eight groups (n = 6). Briefly, category II mice were injected intraperitoneally with potassium oxonate (PO) (250 mg kg−1) 1 h before the final tested drug administration for three consecutive days to increase the serum urate levels. The negative control group (GI) received vehicle (CMC at 0.8%) while hyperuricemic control group (GII) received PO. The positive control group (GIII), which included normal or hyperuricemic mice, received an oral dose of 10 mg kg−1 b.w. of DPI. Groups IV and V received 100 and 200 mg kg-1 b.w. of F. angustifolia leaf ethyl acetate extract (LFA), or 100 and 200 mg kg−1 bark aqueous ethyl acetate extract (BFA). Groups VI, VII, and VIII received 10 mg kg−1 of rutin, luteolin, and esculin, respectively. The animals were sacrificed and the whole blood samples were collected 1 h after the final drug administration. The serum obtained after blood centrifugation (5000 rpm for 10 min) was stored at −20 °C until use. Determination of uric acid levels was achieved using standard diagnostic kits (Spinreact).

### 2.8.2. Assay of xanthine/NADH XOR activity

After the sacrifice, the liver of animals was rapidly excised, washed in saline (0.9%), and homogenized in 5 volumes of 80 mM sodium pyrophosphate buffer (pH 7.4) at 4 °C. The homogenate was centrifuged at 5000 × g for 10 min and the resulting supernatant fraction was further centrifuged at 5000 × g for 15 min at 4 °C. The final supernatant was stored at −80 °C.

The activity of XDH was assayed by monitoring uric acid formation using a spectrophotometric method described previously (Kong et al., 2002) . Briefly, 100 µL of supernatant was added to 50 mM phosphate buffer (pH 7.5), 200 µM NAD+, and 1 mM potassium oxonate to prevent oxidation of uric acid to allantoin, in a final reaction volume of 5 mL. After incubation for 15 min at 37 °C, the reaction was initiated by the addition of 50 mM xanthine. Ten minutes later, the reaction was stopped by the addition of 0.5 mL HCl (0.58 M). Absorbance was recorded at 295 nm for xanthine oxidase activity and at 340 nm for NADH oxidase activity following centrifugation (5000 × g for 5 min). XDH activity was expressed as nanomoles uric acid produced per min per mg protein (nmol UA min−1 mg−1 prot) based on a calibration curve for uric acid. The total protein concentration of the homogenates was determined by the Bradford method (1976) using bovine serum albumin as the standard.

### 2.9. Statistical analysis

The data were expressed as mean ± SD of triplicate assays for in vitro assays and mean ± SEM for in vivo assays. Statistical analysis was carried out using the Graph Pad Prism software (one-way analysis of variance ANOVA). IC50 values were calculated using the OriginPro7.5 software. The differences were considered significant at *P < 0.05, **P < 0.01, and ***P < 0.001.

## 3. Results

### 3.1. Solvent extraction yields and quantification of total phenols, flavonoids, and tannins

Extraction yields from the raw plant material showed a higher percentage for the crude extract of leaves (17%), compared to its bark counterpart (5%) (Table 1). Moreover, a higher yield in leaves was obtained in ethyl acetate phase (11%) while bark constituents were more soluble in the aqueous phase of ethyl acetate (3%).

**Table 1:** Extraction yields, total phenols, flavonoids, tannins, and inhibition of NADH oxidase activity of F. angustifolia extracts from leaves and bark obtained by sequential extraction.

Extracts	Yield (%)		Total phenols(mg VAE g−1)		Flavonoids(mg RE g−1)		Tannins(mg TAE g−1)		Inhibition of NADHactivity (%)	
	Leaves	Bark	Leaves	Bark	Leaves	Bark	Leaves	Bark	Leaves	Bark	
Ethanolic	17	5	536 ± 1	83 ± 1	31 ± 1	63 ± 2	159 ± 3	394 ± 49	73***	85***	
Ethyl acetate	11	0.7	27 ± 1	189 ± 1	46 ± 1	43 ± 1	327 ± 8	66 ± 5	87	86***	
Aqueous ethyl acetate	6	2.85	529 ± 1	68 ± 1	31 ± 1	39 ± 2	66 ± 1	23 ± 23	63***	91*	
Chloroform	10	0.5	389 ± 1	170 ± 1	35 ± 1	55 ± 2	313 ± 6	877 ± 17	80*	80***	
Aqueous chloroform	0.3	0.05	1144 ± 1	25 ± 1	30 ± 1	62 ± 2	57 ± 5	45 ± 44	68***	73***	
Esculin											86***

Data are presented as means ± SD, n = 3. One-way ANOVA followed by Dunnett multiple comparison test was used for statistical significance. *P < 0.05; **P < 0.01; ***P < 0.001 when compared with normal control values. ANOVA = Analysis of variance; SD = standard deviation; VAE = Vanillic acid equivalent per gram of extract; TAE = Tannic acid equivalent per gram of extract; RE = Rutin equivalent per gram of extract.

The quantification of total phenols indicated that the crude ethanolic leaf extract contained significantly more total phenols (535 mg VAE g−1) than the corresponding bark extract (83 mg VAE g−1) (Table 1). The amount of total phenols ranged from 326 to 1143 mg VAE g−1 extract for leaves, the highest being that of the aqueous chloroform extract phase (1144 mg VAE g−1 extract). On the other hand, lower ranges were noticed for bark extracts (25 to 189 mg VAE g−1), the most prominent being that of the organic ethyl acetate phase (189 mg VAE g−1 extract).

Concerning flavonoids, higher amounts were found in extracts of bark in contrast to leaves (Table 1), except for the ethyl acetate extract phase where comparable amounts were observed in both parts of the plant (46 ± 1 and 43 ± 1 mg RE g-1 for leaves and bark, respectively).

### 3.2. Inhibition of NADH oxidase activity of xanthine dehydrogenase (XDH)

The results illustrated in Table 1 indicated that the ethyl acetate extract of leaves (LFA) and aqueous ethyl acetate of bark (BFA) were the most potent against the NADH oxidase activity of XDH (86%; IC50 = 38.51 µg mL−1, 91%; IC50 = 42.04 µg mL−1, respectively), with higher eficiency for leaf extract (P < 0.05). Rutin and esculin suppressed the enzyme as eficiently as LFA and BFA (89%, IC 50 = 32.20 µg mL−1, 86.42%, IC50 = 39.26 µg mL−1) but less than DPI (93%, IC50 = 28.24 µg mL−1).

### 3.3. Correlation analysis

In order to establish a relationship between the amounts of total polyphenols, flavonoids, and tannins and the NADH-inhibitory activity of XOR, a correlation study was conducted. For the most active extract of leaves (LFA), strong correlations were observed between the inhibition of NADH oxidase activity and total phenols (P < 0.001, R2 = 0.92), flavonoids (P < 0.01, R 2 = 0.73), and tannins (P < 0.001, R2 = 0.78) (Figures S6, S8, S10). On the other hand, bark extract (BFA) showed an important correlation between NADH oxidase inhibitory activity and total phenols (R2 = 0.62), but more moderate regarding flavonoids (P < 0.01, R 2 = 0.50) and tannins (P < 0.001, R2 = 0.44) (Figures S7, S9, S11).

**Figure 1:**
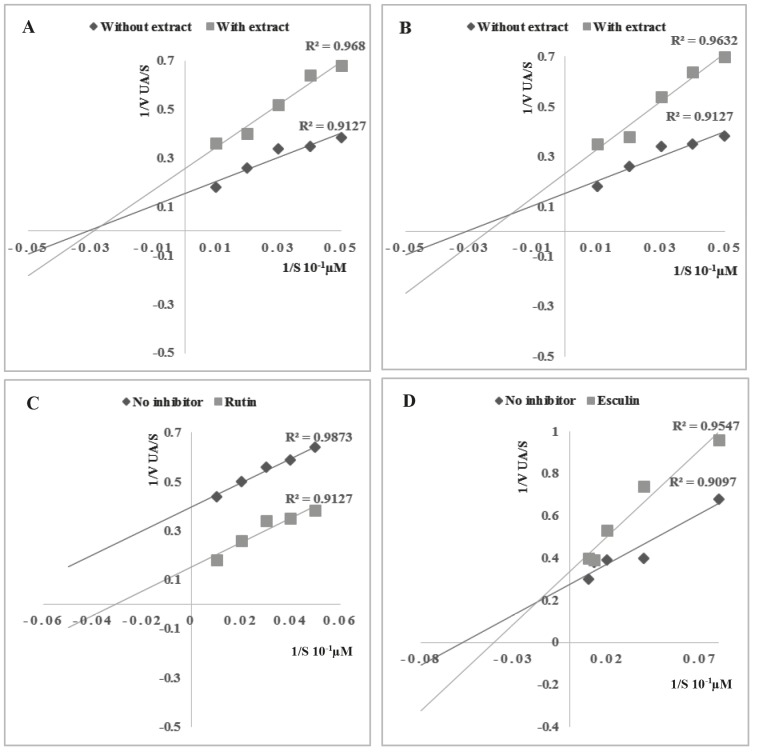
Lineweaver Burk representation of the inhibition of the NADH oxidase activity of XDH by ethyl acetate leaves (LFA) (A) and aqueous ethyl acetate bark (BFA) (B) extracts of F. angustifolia, rutin (C) and esculin (D).

The Lineweaver–Burk plots in the presence of 100 µg mL−1 of LFA or BFA (Figure 1) suggest a mixed-type inhibition. The Km value for leaves was lower than that of bark (Table 2), the latter being comparable to that of rutin (Km = 50 µM). On the other hand, esculin had the lowest Km value (16.66 µM).

**Table 2:** Kinetic parameters of ethyl acetate leaves and aqueous ethyl acetate bark extracts of F. angustifolia on NADH oxidase activity.

	Km (µM)	Km’ (µM)	Vmax (Δabs S−1)	Vmax’ (Δabs S−1)
Leaves	40	152	7.7	2.0
Bark	47	133	10	3.6
Rutin	50	8	0.2	0.03
Esculin	16.66	49.98	4	1.33

### 3.4. Superoxide anion scavenging activity

**Figure 2:**
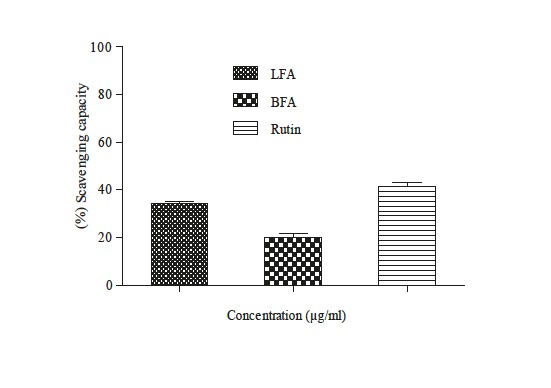
Scavenging activity of superoxide anion generated by PMS-NADH by F. angustifolia extracts, and rutin.

The results illustrated in Figure 2 indicated that both LFA and BFA showed a moderate effect (34% and 19% at 100 µg mL-1, respectively) on the neutralization of the superoxide radical using the PMS-NADH system, lower than their activity in the enzyme (NADH-XDH) system (P < 0.001) (53.69% and 19.17%, respectively) (Table 3).

**Table 3:** Superoxide production and inhibition of superoxide anion generated by NADH – XDH system by F. angustifolia extracts.

Extracts (100 µg mL−1)	Superoxide production(mM) ± SD	Superoxide inhibition(%) ± SD
Ethyl acetate of leaves	0.016 ± 0.02	53.69 ± 0.07
Aqueous ethyl acetate of bark	0.028 ± 0.07	19.17 ± 0.02
Rutin	0.019 ± 0.001	44.1 ± 0.001

Rutin, used as the reference molecule, showed a similar inhibition percentage in the first system of 41% (Figure 2), slightly lower than that of the second (44.1%) (Table 3).

### 3.5. HPLC analysis

HPLC analysis revealed a diverse range of phenolic compounds including lignans, secoiridoids, and flavonoids, with a higher number in bark rather than in leaves (Tables 4 and 5). Data also indicate that some phenol constituents such as verbascoside, calcelarioside, and ligstroside, characteristically found in all Fraxinus species, were shared by the parent crude extract and its derivatives.

**Table 4:** Identification of phenolic compounds in ethyl acetate leaves extract of F. angustifolia using retention times, LC-MS and UV data.

Peak	RT (min)	UV max(nm)	m/z		Aires	(mg eq quercetin kg−1 extract)	Identification
			[M-H−]	MS/MS−			
1	1.5	280	153	123	11.59	154.12 ± 3.5	Hydroxytyrosol (Niemetz and Gross, 2001)
2	4.7	360-260	609	301-208-146-273-343	45.49	253.40 ± 2.5	Quercetin rutinoside(Niemetz and Gross, 2001)
3	4.9	340-285280	463623	301-162461-162	29.74	207.28 ± 2.5	Quercetin glucoside (Niemetz and Gross, 2001) Verbascoside (Sun et al., 2015)
4	5.1	340	593	285-162	4.7	133.9 ± 1.06	Kaempferolrutinoside (Eyles et al., 2007)
5	5.3	340-360	447	285-447	1.25	123.8 ± 0.68	Kaempferol glucoside (Eyles et al., 2007)
6	5.8	280	539	275-307-377-232	143.14	539.3 ± 19.2	Oleuropein (Sun et al., 2015)
7	6.3	280	523	291-223	9.21	147.1 ± 1.02	Ligstroside (Sun et al., 2015)

**Table 5:** Identification of phenolic compounds in aqueous ethyl acetate of bark extract of F. angustifolia using retention times, LC-MS and UV data.

Peak	RT (min)	UV max(nm)	m/z		Aires	(mg eq quercetin kg−1 extract)	Identification
			[M-H−]	MS/MS−			
1	1.4	285	375	152-122-167	62.12	78.5 ± 7.37	Unknown
2	2.2	340	339	177	80.97	92.8 ± 3.33	Esculin (Frison-Norrie and Sporns, 2002)
3	2.7	265	417	161-368-207	174.39	163.9 ± 14.9	Unknown
4	3.4	285-340	369	206-162	263.07	231.4 ± 10.2	Fraxin (Frison-Norrie and Sporns, 2002)
5	3.8	330	429	208-162-221-383-369-206	29.94	54.02 ± 4.29	Unknown
6	3.8	285-340	429	206-177-383-223	41.48	62.8 ± 5.09	Unknown
7	4.5	280	535	373	41.83	63.07 ± 4.5	Pinoresinol(Frison-Norrie and Sporns, 2002)
8	4.6	285-328	477	161-315	60.63	77.3 ± 14.7	Calcelarioside(Frison-Norrie and Sporns, 2002)
9	4.9	280-330	623	461-162	49.30	68.75 ± 9.1	Verbascoside (Sun et al., 2015)
10	5	285-328	477	133-161-315	186.38	173.08 ± 1.3	Calcelarioside(Frison-Norrie and Sporns, 2002)
11	5.1	280-328	685	291-361-523	91.73	101.04 ± 6.7	Ligstroside hexoside (Sanz et al., 2012)

Indeed, our results (Table 5) revealed that the bark of F. angustifolia contained high amounts of both esculin and fraxin (92 and 213 mg eq quercetin kg−1 extract, respectively). On the other hand, the leaves of F. angustifolia were found to be an important source of Kaempferol rutinoside and quercetin glucoside (133.95 and 207.28 mg eq quercetin kg−1 extract).

### 3.6. Characterization and identification of bioactive compounds

**Figure 3:**
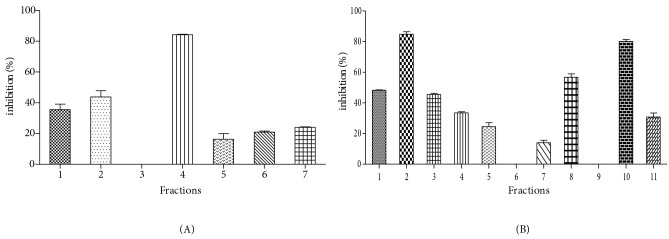
Inhibition of the NADH oxidase activity of XDH by HPLC fractions obtained from ethyl acetate leaves (LFA) (A), aqueous ethyl acetate bark (BFA) (B) extracts of F. angustifolia.

Seven (1–7) fractions were obtained by preparative HPLC chromatography from LFA (Figures S1 and S2) that exhibited respective inhibition rates against the NADH oxidase activity of XDH from 0 to 84.20 ± 0.35 (Figure 3A), while the BFA was fractionated to eleven molecules (1–11) (Figures S3 and S4), showing inhibition rates from 0 to 84.74 ± 1.60 (Figure 3B), respectively.

## 3.7. Antihyperuricemic effect of F. angustifolia extracts in potassium oxonate-induced hyperuricemic mice

### 3.7.1. Uric acid levels

For the uric acid assay, the intraperitoneal injection of PO at a dose of 250 mg kg−1 significantly (P < 0.05) raised the mean serum uric acid levels from 3.16 mg dL−1 (Table 6) to 4.67 mg dL−1 (Table 7). On the other hand, in nonhyperuricemic mice, as illustrated in Table 6, treatment with LFA and BFA (100 mg kg−1) reduced the serum uric acid levels equally and considerably by 40% and 42%, respectively, which is significantly lower than that of the reference drug, DPI (89.9%), in agreement with in vitro results.

**Table 6:** Effects of F. angustifolia extracts and DPI on serum uric acid levels, Xanthine dehydrogenase and on NADH oxidase activities in normal mice.

Groups	Treatment	Dose (mg kg−1 b.w.)	Uric acid reduction(%)	Serum uric acid levels (mg dL−1)	XDH (nmol uric acid mg−1 protein)	XDH Inhibition (%)	NADH oxidase (Umg-1 protein)	NADH oxidaseInhibition (%)
GI	Negative control	-	-	3.1 ± 0.03***	-	-		
GII	Hyper uricemic control	-	-	4.6 ± 0.04***	-	-		
GIII	DPI	10	88.89	0.35 ± 0.003	0.91 ± 0.14	50.26	4.77 ± 0.002	69.57
GIV	LFA	100	40.18	1.9 ± 0.04***	1.32 ± 0.07	21.77	3.02 ± 0.1***	72.92
GIV	LFA	200	34.49	2.07 ± 0.03***	2.05 ± 0.06***	17.52	4 ± 0.14***	64.23
GV	BFA	100	42.08	1.8 ± 0.01***	1.79 ± 0.01***	33.52	6.3 ± 0.002***	59.88
GV	BFA	200	30.69	2.19 ± 0.01***	1.97 ± 0.28***	11.10	5.52 ± 0.04***	59.06
GVI	Rutin	10	-	-	1.83 ± 0.03***	25.13	3.95 ± 0.09***	67.74
GVII	Luteolin	10	-	-	1.26 ± 0.06	29.21	5.07 ± 0.06***	48.66
GVIII	Esculin	10	84.91	0.54 ± 0.01	1.30 ± 0.08	41.29	6.01 ± 0.07***	54.36

Data are presented as means ± SEM, n = 6. One-way ANOVA followed by Dunnett multiple comparison test was used for statistical significance. *P < 0.05; **P < 0.01; ***P < 0.001 when compared with normal control values. LFA = F. angustifolia leaf ethyl acetate extract; BFA= F. angustifolia bark aqueous ethyl acetate extract; ANOVA = analysis of variance; SEM = standard error of the mean. Animal groups are as follows: Group I, negative control; Group II, hyperuricemic control; Group III, diphenyliodonium (DPI) treated group; Group IV, 100 mg kg−1 b.w. F. angustifolia LFA-treated group; 200 mg kg−1 b.w. F. angustifolia LFA extract-treated group; Group V, 100 mg kg−1 b.w. F. angustifolia BFA extract-treated group; 200 mg kg−1 b.w. F. angustifolia BFA-treated group; Group VI, 10 mg kg−1 rutin-treated group; Group VII, 10 mg kg−1 luteolin-treated group; Group VIII, 10 mg kg−1 Esculin-treated group.

**Table 7:** Effect of F. angustifolia extracts and DPI on serum uric acid levels, xanthine dehydrogenase, and on NADH oxidase activities in mice pretreated with the uricase inhibitor, potassium oxonate.

Groups	Treatment	Dose (mg kg−1 b.w.)	Uric acid reduction (%)	Serum uric acid levels (mg dL−1)	XDH (nmol uric acid mg−1 protein)	XDH Inhibition (%)	NADH oxidase (U mg−1 protein)	NADH oxidase Inhibition (%)
GI	Negative control	-	-	3.16 ± 0.03***	-	-		
GII	Hyper uricemic control	-	-	4.67 ± 0.04***	-	-		
GIII	DPI	10	78.84	0.56 ± 0.05	1.54 ± 0.11	48.35	2.69 ± 0.03	72.12
GIV	LFA	100	56.95	2.01 ± 0.04***	2.02 ± 0.24	38.59	1.9 ± 0.002***	75.43
	LFA	200	57.10	2 ± 0.01***	2.87 ± 0.013*	11.86	3.25 ± 0.08***	60.34
GV	BFA	100	57.38	1.9 ± 0.001***	1.85 ± 0,78	36.84	4.68 ± 0.08***	51.32
	BFA	200	53.10	2.32 ± 0.03***	2.99 ± 0.13*	23.21	6.05 ± 0.1***	49.2
GVI	Rutin	10	-	-	1.49 ± 0.046	40.43	4.03 ± 0.1***	59.73
GVII	Luteolin	10	-	-	2.97 ± 0.048*	30.75	4.46 ± 0.05***	52.69
GVIII	Esculin	10	78.94	0.78 ± 0.02	1.10 ± 0.03	49.98	3.18 ± 0.03***	68.27

Data are presented as means ± SD, n = 3. One-way ANOVA followed by Dunnett multiple comparison test was used for statistical significance. *P < 0.05; **P < 0.01; ***P < 0.001 when compared with normal control values. LFA = F. angustifolia leaf ethyl acetate extract; BFA = F. angustifolia bark aqueous ethyl acetate extract. Animal groups are as follows: Group I, negative control; Group II, hyperuricemic control; Group III, diphenyliodonium (DPI) treated group; Group IV, 100 mg kg−1 b.w. F. angustifolia LFA-treated group; 200 mg kg−1 b.w. F. angustifolia LFA extract-treated group; Group V, 100 mg kg−1 b.w. F. angustifolia BFA extract-treated group; 200 mg kg−1 b.w. F. angustifolia BFA-treated group; Group VI, mg kg−1 rutintreated group; and Group VII, 10 mg kg−1 luteolin-treated group; Group VIII, 10 mg kg−1 Esculin-treated group.

In contrast, in hyperuricemic mice (Table 7), the reduction in uric acid levels was more pronounced but followed the same trend with, once again, comparable eficiency for the two extracts (56.95% and 57.38% for LFA and BFA, respectively). A remarkable drop of the amount of blood urate was also noticed for DPI (78.84%), which confirms its hypouricemic effect. Esculin reduced the serum uric acid levels by 84.91% and 78.94% in normal and hyperuricemic mice, respectively (Tables 6 and 7).

### 3.7.2. XDH and NADH oxidase assays in vivo

In order to perform a detailed investigation of the hypouricemic effect of F. angustifolia extracts, liver XDH activities in the presence of xanthine and NADH in normal and hyperuricemic mice were calculated from a calibration curve using uric acid (Figure S5). The results (Table 7) revealed that LFA and BFA (100 mg kg−1) inhibited liver XDH activity effectively and equally (38.6% and 36.8%, respectively) in hyperuricemic mice treated with OP. On the other hand, even though the inhibitory values of the same extracts in normal mice against XDH turned out to be weak, with 21.7% and 33.5% of inhibition, respectively, at the same concentration, they exerted a good NADH oxidase inhibitory activity (72.92% and 59.88%, respectively) in normal (Table 6) and hyperuricemic (75.32% and 51.32%, respectively) mice (Table 7). Moreover, the administration of DPI led to better liver XDH inhibitory activity than the extracts with comparable inhibition in normal and hyperuricemic mice (50% and 48% respectively). Concerning anti-NADH oxidase activity, LFA outperformed DPI, rutin, and esculin (69.57, 67.74 and 54.36%, respectively) in normal (Table 6) and hyperuricemic (72.12, 59.73 and 68.27%, respectively) (Table 7) mice.

The suppression of the orally administered rutin and esculin (10 mg kg-1), two essential components of F. angustifolia, of XDH activity in normal mice was by 25.13% and 41.29%, respectively, compared to oxonatepretreated mice (40.43% and 49.98%, respectively) (Tables 6 and 7, respectively).

## 4. Discussion

The extraction process is a key step that aims to draw out the maximal amount of compounds from the plant. The highest extraction percentages were recorded in the crude extracts of both leaves and bark, with leaves surpassing bark. The results also indicated that the yields of extraction varied considerably between the aqueous and organic phases as a function of the solubility of the plant constituents in the solvent used, as previously observed for other natural extracts (Naczk and Shahidi, 2004) . The organic phase of ethyl acetate of leaves (LFA) marked a high percentage of extraction relatively to the other phases. Moreover, tannins were more abundant in organic phases than in the aqueous ones, implying that they are condensed rather than hydrolysable tannins.

In an attempt to establish a potential relationship between NADH oxidase inhibitory activity and the amount of phenolic compounds in various extracts tested, total phenols, flavonoids, and tannins were determined. Substantial amounts of phenolic compounds were found in all leaf extracts compared to their bark counterparts, which witnesses that F. angustifolia leaves constitute a rich source of bioactive molecules. Moreover, a recent study conducted on F. angustifolia native of Béja (17.55 and 24.84 mg GEA g-1 , respectively) and Nefza (21.86 and 22.85 mg GEA g-1, respectively), two regions in Tunisia, revealed a lower proportion of phenols in leaves compared to bark (Touhami et al., 2017) , compared to LFA and BFA extracts used in the present study. This variation is generally attributed to climatic changes and geographic localization. Higher phenols in the aqueous phase of chloroform derived from leaves reveal a higher amount of constituents with OH groups in this extract (Veličković et al., 2007) . On the other hand, the highest amount of flavonoids in the ethyl acetate phase of leaves is in agreement with previous ifndings (Atmani et al., 2009; Berboucha et al., 2010) and reflects the higher extraction yield of this phase. In fact, a positive correlation between total phenols and inhibition performance of LFA and BFA (Table 1) emphasizes the impact of quantity over performance. On the other hand, a lower correlation was found between the content of flavonoids and tannins and the inhibition of the NADH oxidase activity of XDH.

As a potential source of ROS, the NADH oxidase activity of XDH is considered to be responsible for producing superoxide faster (Sanders et al., 1997) and more (Maia et al., 2007) than its XO counterpart, which highlights the importance of its inhibition. However, this activity has been little studied, which motivated the present investigation. Our interest focused on the attenuation of NADH oxidase by extracts of F. angustifolia to assess their capacity to prevent oxidative stress-related pathologies. Moreover, the traditional use of F. angustifolia against gout drew our attention to their possible involvement in XOR inhibition. LFA was more potent against the NADH oxidase activity of XDH than BFA, but both remained less eficient than DPI. A previous report (Atmani et al., 2005) indicated that DPI (1 µM) strongly inhibited the NADH oxidase activity of caprine milk XOR (97.3%), a result narrowly close to our findings concerning this molecule. The specific structure of DPI, an aromatic heterocyclic cation composed of 2 benzene rings fused with an iodolium ion, seems to be ideal for the inhibitory activity of the FAD site of the enzyme.

In an attempt to gain more insight into the mechanism of action of F. angustifolia extracts, NADH oxidaseinhibitory activity of XDH was tested using various concentrations of NADH in the absence and presence of selected extracts and standard. A mixed-type inhibition was recorded which is a predictable outcome pertaining to the fact that an extract is not a pure molecule but rather a mixture of components exerting different actions on the enzyme, some being competitive inhibitors, others noncompetitive. On the other hand, rutin, an important constituent of leaves (Table 2), showed uncompetitive inhibitory activity (Figure 1), whose action could be altered by other active components of the extract, thus leading to a mixed-type inhibition for that extract. The same applies to esculin, which showed a noncompetitive mixed inhibitory activity (Figure 1).

The efficiency of the extracts on the repression of XDH could be explained by the nature of their constituents, which could exert an inhibition on the active sites of the two forms of the enzyme, but more importantly on the FAD site where substrates of XDH favorably act. In fact, the conversion of XO to XDH results in conformational changes affecting mostly the FAD site (Enroth et al., 2000). Bindoli et al. (1985) revealed that different types of inhibition of the oxidase (competitive type) and the dehydrogenase (mixed-type) appear to depend on the redox state of the sulfhydryl groups of xanthine-oxidizing enzyme. This means that in the dehydrogenase form, reduction with DTT favors a conformational modification of the enzyme (more dithiols over the disulfides), hence affecting its kinetic parameters. A previous study on XO/ xanthine inhibition by F. angustifolia extracts (Berboucha et al., 2010) demonstrated that the ethyl acetate leaves extract and its aqueous counterpart of bark exerted less inhibition on XO, compared to XDH, in close agreement with Bindoli’s observations. In the same context, allopurinol (5 µM), oxypurinol (5  µM), and amufltizole (1 µM), which are drugs often used to combat gout and arthritis and reputed to be strong inhibitors of xanthine oxidase activity of XOR, poorly suppressed the NADH oxidase activity of XOR (Atmani et al., 2005), which confirms that the two forms of the enzyme have two different sites of inhibition.

The comparison of Km values gives an indication of the affinity between substrate and inhibitor of the enzyme (Dixon, 1972). The lower the Km, the higher the affinity of inhibitor to substrate-enzyme complex. Indeed, the Km values of the leaves and bark extracts (Table 2) are in satisfactory agreement with their high inhibitory activity obtained against the NADH oxidase activity of XDH, while the Km value for esculin was the lowest, demonstrating its stronger affinity to substrate-enzyme complex.

It has long been recognized that naturally occurring substances in higher plants were endowed with antioxidant activity (Atmani et al., 2009) . The most prominent one, flavonoids, are reputed to be powerful free radical scavengers. Superoxide anion is produced by NADH oxidase activity (Sanders et al., 1997; Atmani et al., 2005) and can be a source of many deleterious free radicals such as OH. (Doroshow, 1983). The potential elimination of this radical can occur through simple scavenging (nonenzymatically) or by inhibition of the NADH oxidase activity of XOR. Both methods were assessed and concluded to have the better performance of the extracts in the enzymatic system, which is in accordance with their high anti-NADH oxidase activity of XDH, as shown above. Since the nonenzymatic system evaluates solely the scavenging activity of the extracts while the enzymatic system is a result of both scavenging and enzymatic inhibition, we can infer that the suppression of the generation of superoxide radical by the NADH oxidase activity of XDH has overridden that of its scavenging potential. Our findings also confirm a higher potential for LFA to suppress the production of superoxide enzymatically, which is in agreement with its higher NADH oxidase inhibitory activity. These results are highly relevant in relation to the fact that superoxide anion is implicated in endothelial dysfunction and therefore in the genesis of cardiovascular disease (Hamilton et al., 2001) .This was demonstrated for DPI, which, as it suppressed NADH oxidase activity strongly thereby eliminating superoxide anion, led to the improvement of endothelial function (Hamilton et al., 2001) . On the other hand, the results obtained for rutin reflect its NADH oxidase inhibition potential (Figure 2), but also reveal a high scavenging activity, in contrast to those of extracts.

In order to characterize the bioactive compounds responsible for the anti-NADH oxidase activity, the HPLC analysis was carried out. The characterization of all the extracts was based on the comparison of UV data, mass spectrometry (MS), and MS fragmentation behavior with the published data standards. We focused our attention on the most active extracts, namely LFA and BFA, in order to identify their most active constituents. It should be noted that ligstroside hexoside was identified for the first time in the composition of F. angustifolia bark while previous investigations detected this compound in Fraxinus excelsior (Iossifova et al., 1997) . Furthermore, we noticed the occurrence of rutin in leaves and its absence in bark (Table S1 and S2), as previously reported (Ayouni et al., 2016; Medjahed et al., 2016). Our data have also confirmed a high proportion of hydroxycoumarin glucoside (esculin) in both leaves and bark of F. angustifolia, an compound which is considered to be a characteristic feature of Fraxinus species (Jenson et al., 2002; Perez et al., 2005) .

The coumarin glucosides esculin and fraxin occur in almost all Fraxinus species. However, their ratio varies depending on the different plant sources. For example, esculin predominates in Fraxinus ornus bark (Kong et al., 2002) . The presence of Kaempferol rutinoside and quercetin glucoside in F. angustifolia leaves was confirmed by a metabolomic study (Ayouni et al., 2016) , which showed that flavonoid glucosides (Kaempferol 3-O-rutinoside, quercetin 3-O-glucoside, and rutin) were revealed to be highly involved in the antioxidant potential of leaves.

LFA and BFA, being the most active against NADH oxidase activity, were further fractionated by preparative chromatography. A higher number of compounds in bark reassert the wider diversity of polyphenols in that part of the plant rather than leaf extracts. Moreover, more flavonol glycosides such as rutinoside derivatives of quercetin and kaempferol were detected in ethyl acetate extract of leaves rather than in the corresponding aqueous phase of ethyl acetate of bark. Previous investigations reported that lfavanols having a 7-hydroxyl group such as quercetin are included among the most effective inhibitors of XDH (Zhu et al., 2004), which is in line with the higher inhibition potential of leaf extracts.

**Figure 4:**
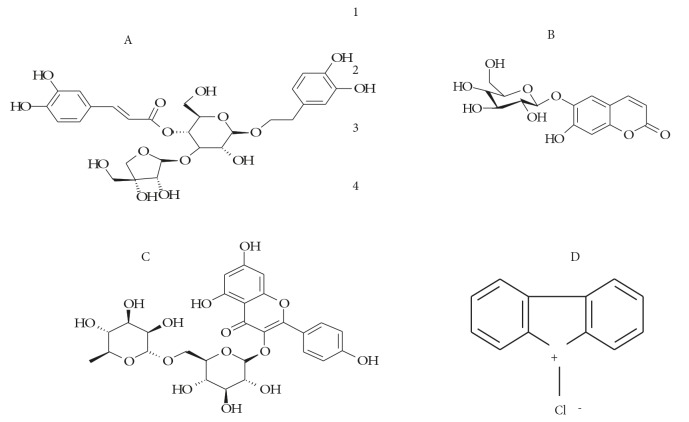
Structure of calcelarioside (A), esculin (B), kaempferol rutinoside (C), and DPI (D).

In fact, the highest inhibition rate for leaves was exhibited by fraction 4 containing kaempferol rutinoside (Table 4). Esculin, on the other hand, was identified in the most potent fraction 2 of bark, followed by fraction 10 containing calcelarioside (Table 5). These compounds were not found in their highest proportion in the extracts, in contrast to oleuropein and fraxin, which were found in considerable amounts in leaves and bark, respectively (Tables 4 and 5). These observations highlight the fact that the quantity of the constituents may not be as important as their structure in their inhibition potential against the NADH oxidase of XDH. This is in close agreement with the low correlation of flavonoids with activity. Indeed, if the structural features of kaempferol rutinoside and esculin are examined, it can be noticed that they share a high number of OH groups, with a catechol moiety, features that correspond highly to a structural inhibitor of xanthine oxidase, allopurinol. However, considering the fact that allopurinol is a poor inhibitor of the NADH oxidase activity of XDH from caprine milk (14.2% at 5 µM) (Atmani et al., 2005) , we decided to compare the structure of calcelarioside kaempferol rutinoside and esculin with DPI (Figure 4).

The striking structural characteristic of DPI is its symmetry and the presence of a catechol moiety, the latter being shared by the three most active compounds. Both calcelarioside and esculin also have a glucosyl moiety in common. This study was preceded by a previous report demonstrating that esculin was a moderate inhibitor of xanthine oxidase in vitro (Chang and Chiang, 1995), in contrast to its high bioactivity against the NADH oxidase of XDH demonstrated in this study, which confirms again the different sites of inhibition of the two forms of XOR.

Therapeutic strategy against hyperuricemia is mediated through the inhibition of XOR by allopurinol or related drugs. Although they greatly reduce uric acid production, these molecules are highly toxic. Therefore, the discovery of safe, novel, and natural lead molecules that perform the same functions would give hope for patients with hyperuricemia. In the present report, an in vivo experiment was conducted to tackle the disparity issue between in vitro and in vivo results. The latter (Tables 6 and 7), which depicted the levels of uric acid, NADH oxidase, and XDH inhibitory activities of the extracts in normal and hyperuricemic mice, respectively, as well as DPI, were shown to possess potent hypouricemic effects in both cases. In fact, intraperitoneal injection of OP elevated the mean levels of serum uric acid. This is due to the inhibitory effect exerted by oxonate on uricase, which converts uric acid to allantoin.

On the other hand, LFA, BFA, DPI, and esculin reduced the serum uric acid levels in normal and hyperuricemic mice, which ascertains their hypouricemic potential. Esculin may be responsible for the hypouricemic effect of bark by upregulating the expressions of renal organic anion transporter 1 (mOAT1) and organic cation and carnitine transporters (mOCT1-2 and mOCTN1-2), as explained by Li et al. (2011) .

The inhibition of liver XDH activity in hyperuricemic mice by LFA and BFA is in accordance with their equal performance in reducing uric acid levels, as reported above.

In a previous study (Zhu et al., 2004), rutin has shown a similar inhibitory potency on XDH activity in hyperuricemic mice while DPI (10 mg kg-1) had the same impact on normal and hyperuricemic mice.

Esculin was found to possess a good hypouricemic potential in rodents, not related to its xanthine oxidase inhibitory activity and only when administered intraperitoneally, indicating that intestinal absorption decreases its availability (Kong et al., 2002; Li et al., 2011) .Our results corroborate the decrease in biodisponibility of esculin in vivo as the reduction in NADH oxidase was diminished (54.36%) (Table 6) compared to in vitro ifndings reported above (86.42%) (Table 1). The good inhibitory impact of extracts, rutin, and esculin on the NADH oxidase activity of XDH (59.73 and 68.27%, respectively) (Table 7) rather than xanthine oxidase may justify their hypouricemic effect.

In conclusion, phenolic compounds are well known antioxidants that attract curiosity for their possible therapeutic use against various disorders and ROSmediated diseases. The high potency of leaf and bark extracts of F. angustifolia against the NADH oxidase activity of xanthine dehydrogenase both in vitro and in vivo provides strong evidence for the use of this plant to fight antiinflammatory disorders, particularly gouty arthritis. Reputed for their large array of biological activities, the identified active constituents of F. angustifolia (kaempferol rutinoside, esculin, and calcelarioside) represent a beneficial therapeutic tool and may well become good candidates for exploitation in the pharmacological industry.

## Acknowledgments

The authors wish to thank the Ministry of Higher Education and Scientific Research of Algeria for sponsorship (Grant number: F00620100006). We also appreciate the precious help of Professor Carlos Gutierrez Merino from Universidad de Extremadura and Alijandro K. Samhan Arias from Universidad Nova de Lisboa, 2829516 Caparica, Portugal, for their valuable advice.
